# Modification Method of High-Efficiency Organic Bentonite for Drilling Fluids: A Review

**DOI:** 10.3390/molecules28237866

**Published:** 2023-11-30

**Authors:** Yi Pan, Xinyue Zhang, Chengcheng Ji, Qianru Zhan, Zhaoxuan Li, Jian Guan, Jian Huang

**Affiliations:** 1 Department of Petroleum and Natural Gas Engineering College, Liaoning Petrochemical University, No. 1, West Section of Dandong Road, Wanghua District, Fushun 113001, China; panyi_bj@126.com (Y.P.); zhagxinyue78@163.com (X.Z.); zqr9702@126.com (Q.Z.); lzx_zj@163.com (Z.L.); 2 Engineering Training Centre, Liaoning Petrochemical University, No. 1, West Section of Dandong Road, Wanghua District, Fushun 113001, China; 3 Engineering Department of Greatwall Well Drilling Company, China National Petroleum Corporation, Panjin 124000, China; gj_04081@163.com; 4 Liaohe Oil Field Construction Company of China National Petroleum Company, Panjin 124000, China; hj_0906@163.com

**Keywords:** modification, intercalation, coupling, grafting, molecular simulation

## Abstract

The requirements for drilling bentonites are tightening due to ever-increasing demands for petroleum resources, coupled with cost and reaction technology constraints. In addition to raising the risk of drilling, bentonite’s poor performance also raises the possibility of safety incidents and significant financial losses. Organically modified bentonites effectively reduce the consumption of drilling fluids, conserve resources, and lessen environmental effects. This paper aims to provide an overview of the several organic modification methods of bentonite for drilling fluids. It also evaluates the characteristics and application impacts of bentonite. We primarily describe the three popular modification methods represented by intercalation, coupling, and grafting. Also, this review provides the effect of molecular simulation on the investigation of structure in microconfined conditions. Through microlearning, organically modified bentonite with exceptional performance is to be further developed.

## 1. Introduction

The main component commonly used in drilling fluids is bentonite. It is widely used in the ceramics industry, oil exploration, wastewater treatment, paper manufacturing, and many other fields [1]. Strong hygroscopicity and swelling properties make it a “universal material”. Bentonite is a type of layered silicate clay mineral mainly composed of montmorillonite. Montmorillonite (hereinafter referred to as Mt) is a layered aluminosilicate. It is composed of one aluminum–oxygen octahedron and two silicon–oxygen tetrahedrons. The hydration ability is strong because oxygen atoms make up the upper and lower crystal layers, and there is a relatively weak connection between them. The interlayer of montmorillonite contains cations, primarily Na^+^ and Ca^2+^; the interlayer spacing is approximately 0.96~4 nm and can be utilized for ion exchange [2,3]. The following characteristics are the key features of montmorillonite: (1) Chargeability. Because of the substitution of the montmorillonite lattice, it has a permanent negative charge. To balance the charge, montmorillonite exchanges ions and binds different cations to alter its properties. (2) Adsorption. The adsorption on the montmorillonite surface is mainly physical adsorption and chemical adsorption. Physical adsorption is primarily produced by intermolecular interactions, and chemical adsorption is mainly produced by chemical bonds, such as covalent bonds. (3) Expansion. Because of its relative hydrophilicity, montmorillonite absorbs water and expands in volume. (4) Cohesion. Montmorillonite can hydrate and expand under specific circumstances; this process has an impact on the viscosity and fluid loss of drilling fluid. The variability (layer charge) of montmorillonite is a direct factor affecting the performance of organic bentonite [4]. The smaller the number of montmorillonite layers, the better the dispersibility in water. In order to achieve a guaranteed modification effect, the inorganically modified montmorillonite needs to ensure total contact with the modified particles (molecules, ions, etc.) during the organic process.

Sodium-based bentonite can be utilized as the main slurry ingredient in drilling fluid. The attributes of sodium-based bentonite include high cost, high water swelling rate, sluggish water adsorption speed, good dispersion in water, and thixotropy. Although China has a large amount of bentonite overall, high-quality sodium-based bentonite is severely lacking. Therefore, it is necessary to carry out sodium-based modification treatment on calcium-based bentonite to improve the utilization rate of related resources.

The inorganic modification of montmorillonite involves replacing the exchangeable cations adsorbed between the layers of montmorillonite mineral crystals through different processes. When the concentration of sodium ions in the dispersion system is high, the exchangeable divalent calcium ions adsorbed between the mineral layers in bentonite are replaced by low-valent sodium ions [5] and thereby converted into sodium montmorillonite. The reaction process is shown in Formula (1):

(1) $$\mathrm{Ca}-\mathrm{Mt}+2{\mathrm{Na}}^{+}\to \mathrm{Na}-\mathrm{Mt}+{\mathrm{Ca}}^{2+}$$

After sodium modification of natural calcium-based montmorillonite, the interlayer domain value of montmorillonite decreases due to the replacement of interlayer calcium ions with sodium ions. In the characterization of the inorganically modified montmorillonite, it was found that the vibration peak of the Si-O-Si framework at 1000–1100 cm^−1^ of the montmorillonite had broadened following the inorganic modification. This finding suggests that the degree of crystallinity and the distribution of the silicate structural force of modified montmorillonite have changed. The degree of structural force change determines the dispersibility of montmorillonite. The higher the dispersion performance, the broader the vibration width of the Si-O-Si framework, suggesting that montmorillonite’s dispersibility is enhanced following inorganic alteration [6].

At present, the modification methods of sodium-based bentonite include the dry process and the wet process.

The dry process refers to adding sodium agent dry powder (commonly Na_2_CO_3_) to bentonite and squeezing the two to fully react. Typical techniques include spiral flow blocking extrusion, double spiral mixed extrusion, wheel grinding, etc. The initial humidity of the dry-modified bentonite directly affects its application impact. Despite the dry process having low energy consumption and a simple process flow, there is no purification operation for the original bentonite. The presence of silicates in the original soil leads to a decrease in the amount of sodium-based bentonite formed, and the resulting bentonite has a relatively high impurity content. Additionally, this process requires operating at high pressure or the surfactant’s melting point, which consumes a lot of energy and cannot ensure the uniformity and stability of the finished product [7].

The wet process is another name for the suspension process. In order to achieve the effects of sodium treatment, bentonite must be prepared into the slurry at a concentration of less than 50%, added in large quantities along with other sodium modifiers, and stirred continuously to expand and disperse the bentonite [8]. Although there are significant challenges in the drying and dehydration process, the wet process can guarantee that the bentonite quality satisfies the specifications for industrial uses. The manufacturing of organic bentonite is more expensive and less suitable for industrial use due to processes like stirring, drying, and activation that need a lot of water and energy, as well as a lengthy preparation period.

Oil and gas production will have some effect on the environment. Consequently, domestic and foreign scholars have proposed many protective measures for the development process, such as CO_2_ hydrate sub-seabed sequestration [9] and high-performance guar gum fracturing fluid to protect storage [10]. Drilling fluids are indispensable in the field of drilling engineering. The development of drilling-fluid materials to meet drilling and environmental requirements has been an ongoing concern [11]. For drilling-fluid systems in oilfields, bentonite has long been utilized as a base mud material, primarily to improve fluid viscosity and to adjust the density [12]. As an important material for drilling fluids, bentonite can be modified to improve resource utilization, ensure the smooth progress of drilling work, and play an important role in the development of related industries.

Drilling fluids are mainly based on dispersed sodium bentonites. The special requirements can only be met by components whose properties may in some cases be counteractive. For instance, the thixotropic property of the fluid requires conditions that conflict with optimal plastering. When calcium and/or barium sulfate are added to enhance the density or calcium and barium hydroxide are added to reduce the sharp increase in viscosity at the high temperatures in deeper holes, the divalent cations cause coagulation of the dispersed particles. This effect is usually overcome by the addition of complex macromolecular compounds that also impart a high degree of steric stabilization. A well-known additive is carboxymethyl cellulose. The properties of bentonite that are most significant in the above-mentioned engineering applications are viscosity, yield value, thixotropy, plastering ability, and plasticity. Thus, only natural sodium bentonites and certain sodium-exchanged calcium (magnesium) bentonites are suited for this purpose.

Due to the excellent properties of montmorillonite, deep processing of modified bentonite can produce organic bentonite. Organic bentonite complexes can be formed by adding organic modifiers to the bentonite layer to increase the spacing of the montmorillonite layers. Long hydrocarbon chains are used to coat the bentonite wafer’s surface, forming organic hydrophobic and hydrophilic oil layers. In order to improve the carrier lithology and rheology of the drilling fluids, prevent corrosion during drilling, and ensure adequate lubrication, organically modified bentonite is used as a viscosity-increasing and shear-lifting agent in drilling fluids. It is widely used in salt rock formations, water-sensitive formations, deviated wells, and high-temperature deep wells.

At present, the development of organic bentonite is mainly based on quaternary ammonium salt modification, and relatively new modifiers include organic acid compounds and phospholipid derivatives. The primary goal of the development direction of organic bentonite research is to develop new modifiers, additives, etc. Parameterization, generality, and convenience should be the direction of current drilling-fluid development.

## 2. Organic Modification Mechanism of Bentonite

The cost of natural bentonite and drilling operations has significantly increased as a result of the increased demand for American Petroleum Institute (API) grade drilling bentonite brought on by oil exploration activities. Different approaches are being used to improve drilling performance [13]. Drilling operations can be made less expensive overall by raising the rate of penetration (ROP) and boosting drilling efficiency [14].

The use of organic bentonites as additives in organic-based drilling fluids has been a research issue. Ionic surfactants are used as rheology control additives in drilling fluids to prevent flocculation of solids such as organic bentonite and to maintain the mud dispersed [15]. In order to insert organic groups into montmorillonite, the principle of organic modification of montmorillonite is to replace the exchangeable cations between the montmorillonite layers with cationic surfactants (e.g., organic amine salts and quaternary ammonium salts), nonionic surfactants, etc. Montmorillonite is hydrophilic due to the presence of inorganic exchangeable cations in the interlayer space and lipophilic due to the organic interlayer components [16].

For decades, this method based on cation exchange with alkyl ammonium ions was used to produce organic bentonite. The alkylammonium species used to prepare organic bentonite are quaternary ammonium compounds containing alkyl, phenyl, benzyl, and pyridyl groups. A mixture of alkyl ions and alkenylammonium ions is used in certain situations. Controlling the rheological behavior in organic solvents, where they function as thickeners and thixotropic agents, is the most significant use of these organic bentonite particles.

As early as the 1930s, it was discovered that organic ammonium (R-NH_3_) could react with sodium bentonite. In 1949, the organic modifier carbon chain was first proposed and various organic bentonite were synthesized. The length has an impact on the modification effect of organic bentonite. The organic bentonite made with CTAB/DTAB was characterized for the first time using XRD and TGA [17]. Despite starting relatively late, China has conducted a large amount of research on organic bentonite in recent years. CTAB was used as a modifier to modify bentonite, and the performance of the modified organic bentonite was evaluated using X-ray diffraction, scanning electron microscopy, and other methods [18]. The organic bentonite was prepared by using modifiers such as TTAB, which showed that the thermal stability of the organic modified bentonite was better than that of the organic bentonite prepared by using CTAB [19]. The modified organic montmorillonite with CTAB was analyzed using X-ray diffraction, scanning electron microscopy, thermogravimetric-differential thermal analysis, and other techniques. The interlayer structure of montmorillonite contained sufficient cations [20]. Organic montmorillonite was prepared using organic cationic modifiers such as CTAB and OTAB, and it was characterized using X-ray diffraction, infrared ray diffraction, and other analytical methods. The experimental results found that the quality of the modified montmorillonite mineral crystal layer improved with lengthening of the modified intercalating agent’s alkyl chain [21]. Using 1827 as a modifier, an orthogonal experimental design is used to examine and talk about the production process of organic montmorillonite. After analysis of the produced products’ gel and structural characteristics, it was discovered that they have good gelation and dispersibility characteristics in organic solvents [22]. CTAB was used as a modifier in the preparation of the organic montmorillonite. The montmorillonite was purified and modified with sodium before the researchers introduced the modifier. The resultant organic montmorillonite product was created by stirring it at a specific temperature. The suspension performance was evaluated, and relatively better conditions were suggested for the modification process [23]. Cationic surfactants and nonionic surfactants were used as modifiers to produce cationic-nonionic organic bentonite, and anionic surfactants and cationic surfactants were used to produce anionic-cationic organic bentonite. Then, the adsorption capacity of the produced organic bentonite towards organic pollutants such as phenol, aniline, etc., was evaluated [24,25]. For bentonite with a greater salinity, organic bentonite was made with the OTAB modifier. The optimal modification conditions were determined by evaluating the viscosity of the prepared organic bentonite, and the prepared organic bentonite was characterized, which indicated low ultraviolet transmittance and good UV resistance [26].

### 2.1. Intercalation

Layered silicate is formed by stacking silicate layers about 1 nm thick, and cations such as sodium, potassium, magnesium, and calcium are generally adsorbed between the silicate layers. These cations can be replaced by other organic macromolecules through an ion exchange reaction (i.e., silicate is treated organically) to increase the interlayer spacing of the silicate layers. Under the action of external factors such as solvents, chemicals, mechanical mixing, and shearing force, the polymer macromolecules are inserted between the silicate layers to overcome the force between the layers. The layers are then peeled off and dispersed in the polymer matrix to produce the polymer complex of layered silicates. [27]. Depending on the degree of dispersion of the silicate layers in the polymer matrix, the composites can be divided into four categories: (1) Incompatible composites. The silicate particles are particularly poorly dispersed in the polymer matrix and have an agglomerated structure. It is incompatible with the polymer matrix. (2) Partially compatible composites. The silicate particles have a certain compatibility with the polymer matrix and are dispersed in the polymer matrix, but the polymer cannot be intercalated into the silicate. The interlayer system has the performance of ordinary polymer-filled particles. (3) Intercalated composites. The polymer can intercalate into the silicate lamellae but cannot overcome the interlayer force of the silicate lamellae [28]. (4) The exfoliated composite. The polymer is completely intercalated in the silicate layer, the order of the silicate layer stack is destroyed, and the exfoliated layer is evenly distributed throughout the polymer [29]. Among them, exfoliated complexes reflect the unique properties of PLSN to the greatest extent possible. Consequently, the goal of any PLSN preparation method’s research and development should be to obtain exfoliated complexes.

The principle of organic modification of bentonite is to replace the exchangeable cations between the bentonite layers with cationic surfactants (e.g., quaternary ammonium salts). As a result, the alkyl chains enter the interlayer and improve the hydrophobicity and interlayer spacing of bentonite. It can be further modified with an alkylsilane coupling agent. The silane can be hydrolyzed, causing dehydration condensation between the silanes and the hydroxyl groups between the bentonite layers and the bentonite surface, resulting in the formation of siloxane covalent bonds, further increasing the dispersion of bentonite. Formulas (2) and (3) are the reaction formulas of organic modification treatment of bentonite:

(2) $$\mathrm{bentonite}\left[X+R-\mathrm{NBr}\right]\to \mathrm{bentonite}\left[R-N^{+}\right]+\mathrm{XBr}$$

(3) $$\left[R-N^{+}\right]\mathrm{bentonite}\left[\mathrm{Si}-\mathrm{OH}\right]+\mathrm{RSi}\left({\mathrm{OCH}}_{3}\right)\to \left[R-N^{+}\right]\mathrm{bentonite}\left[\mathrm{Si}-O-\mathrm{Si}{\left(\mathrm{OH}\right)}_{2}R\right]$$

In Formulas (2) and (3), X is Na^+^, H^+^, and Ca^2+^; R is an alkyl group with sixteen carbon atoms.

Studies have shown that the intercalation of cationic surfactants is related to the length of the alkyl chain [30], with shorter alkyl chains having poor molecular dispersion and longer alkyl chains being difficult to intercalate.

### 2.2. Coupling

At present, the methods used for organic bentonite modification are mainly divided into exchange or adsorption of cations between montmorillonite layers of organic compounds in different environments and surface modification with silane cross-linking agents. The coupling agent is a chemical substance with an amphoteric structure. One part of the molecule’s groups reacts with various functional groups on the silicate surface to form strong chemical bonds, and another part of the groups reacts chemically or physically with organic matter to increase affinity and compatibility. The most recent advancement in petroleum technology and nanotechnology is the use of nanoparticles in oil drilling [31]. The coupling agent is grafted onto the surface of the nanoparticle to form a “dyeing molecule” monomer, and then a copolymerization reaction takes place on the powder’s surface to form a high-molecular-weight layer. The hydroxyl group on the surface of the nanoparticle serves as a reactive group; coupling agent molecules are grafted onto it, and then the polymerization reaction is carried out. This method is generally the first choice for silane coupling agents. A silane coupling agent is an organic compound with double reactive functional groups, polar and non-polar groups, all of which contain silicon or metal atoms. The polar groups in their molecules are hydrophilic and react with the hydroxyl groups on the surface of nanomaterials to form new chemical bonds; at the same time, the non-polar groups in the molecules enter into organic reactions or chain entanglements with the polymers in the system [32].

Silane coupling agents have been widely used to produce micro-composites based on silica, fiberglass, etc. Because these agents make the montmorillonite surfaces organophilic, they are also effectively used to produce nanocomposites. Organosilane coupling agents are able to form stable siloxane bridges by reacting with silanol groups on the montmorillonite surface through the presence of RSi–X groups (X ¼ OR, Cl).

(4) $$\left[\mathrm{surface}\right]\equiv \mathrm{Si}-\mathrm{OH}+X-\mathrm{Si}\equiv \left[R_{1}R_{2}R_{3}\right]\to$$

(5) $$\left[\mathrm{surface}\right]\equiv \mathrm{Si}-O-\mathrm{Si}\equiv \left[R_{1}R_{2}R_{3}\right]+\mathrm{HX}$$

The coupling agent acts as a medium to enhance the interaction between inorganic nanoparticles and organisms. The coupling agent plays an important role in grafting macromolecules onto the surface of nanoparticles. On the one hand, it reduces the hydrophilicity of the particle surface and improves its compatibility with the polymer monomer. For example, the coupling reaction between the coupling agent and the hydroxyl group on the surface can improve the activity of the grafting reaction [33]. On the other hand, the polymer coating of the monomer on the surface of the nano-powder can be encapsulated by microemulsion polymerization. During this process, the monomer is polymerized to form a coating on the surface of the particle.

### 2.3. Graft

The adsorption of neutral molecules on montmorillonite is driven by various chemical interactions: hydrogen bonds, ion–dipole interactions, coordination bonds, acid–base reactions, charge transfer, and van der Waals forces. Polar molecules such as alcohols, amines, amides, ketones, aldehydes, and nitriles form intercalation complexes with smectites. Guest compounds can be intercalated from the vapor, liquid, and solid states. When intercalated from solutions, solvent molecules are generally co-adsorbed in the interlayer space. Due to the use of montmorillonite and polymers in many industrial applications, the interaction of montmorillonite with organic macromolecules has attracted great attention. In many cases, the polymers are adsorbed on the external surface and are not intercalated. Therefore, we use the behavior of polymer adsorption on the surface of montmorillonite to modify the properties of montmorillonite.

Grafting is the process of forming large molecular chains with appropriate branched or functional side groups through chemical bonds under the action of an initiator, resulting in a product called a graft copolymer. Through coupling processes, organic groups such as ethylene or ethylene groups can be grafted and polymerized on the surface of inorganic particles. This process is known as surface grafting. It is simple to graft different vinyl-based polymers onto the surface of inorganic particles [34]. In general, sufficient hydroxyl groups have accessibility, biodegradability, and reaction compatibility with other molecules [35].

Drilling fluids play an important role in oil and gas well drilling operations [36]. The drilling-fluid filtrate reducer is mainly adsorbed on the surface of the bentonite particles to form an adsorption layer. It can not only disperse the aggregation of the bentonite particles but also thicken the hydration film around the bentonite particles to reduce the filter cake. The polymer fluid-loss reducer reduces fluid loss by blocking the entrance of filter cake pores [37]. In the 1970s, researchers started studying the graft copolymerization of starch and vinyl monomers. The reaction principle is as follows: Under the action of the initiator system, the starch-CC-skeleton first generates free radicals, followed by the production of vinyl monomer free radicals. These are grafted onto starch-free radicals to obtain graft copolymers through chain growth. The grafting copolymerization of starch and vinyl monomers must have a process of initiating free radicals, and the grafting copolymerization reaction methods include physical initiation and chemical initiation. The physical initiation method has high initiation efficiency, which mainly includes photoinitiation and radiation initiation. After the reaction is completed, there is no residual initiator or chemical reagent in the product. The chemical initiation process mainly generates free radicals through redox reactions, and the performance of different types of initiators varies greatly. Common initiators include hydrogen peroxide/cerium ammonium nitrate initiation system and the ozone/oxygen mixed system. Under the action of the initiator, the bond between the glucose units C2 and C3 on the starch molecule is oxidatively broken to form free radicals, the starch and vinyl monomer undergo a free-radical polymerization reaction, and the graft copolymer is generated by the starch and the monomer under the action of the initiator. There are two types of polymers: copolymers, which are formed by mutual addition of starch and graft monomers, and homopolymers, which are formed by mutual polymerization of monomers. It is a relatively complex mixing system. Therefore, in order to obtain the target product, the production of homopolymers should be suppressed by optimizing each process.

## 3. Intercalation-Modified Bentonite

The intercalation method uses the unique structure of montmorillonite to intercalate the polymer into its silicate layer structure, destroy its layer structure, and realize the nanometer-scale composite. The literature shows that the rheological properties of organic bentonite improve with increasing interlayer spacing [38]. There are two types of polymer intercalation: melt intercalation and solution intercalation [39]. In general, the composites obtained by polymer solution intercalation and polymer melt intercalation have the same structure.

### 3.1. The Method of Solution Intercalation

The macromolecular polymer chain is intercalated into the silicate layer using a solvent in the solution in the polymer solution intercalation method. The solvent is then volatilized to achieve the polymer’s insertion into the interlayer. The ion-exchanged bentonite and the polymer solution are mixed and stirred uniformly, and then the solvent is removed by evaporation. Bentonite and additional additives are uniformly mixed with water during polymer solution intercalation, and polymer–bentonite nanocomposites are subsequently prepared by drying and washing. This method requires finding a suitable solvent to dissolve the polymer and bentonite at the same time [40].

**Figure 1:**
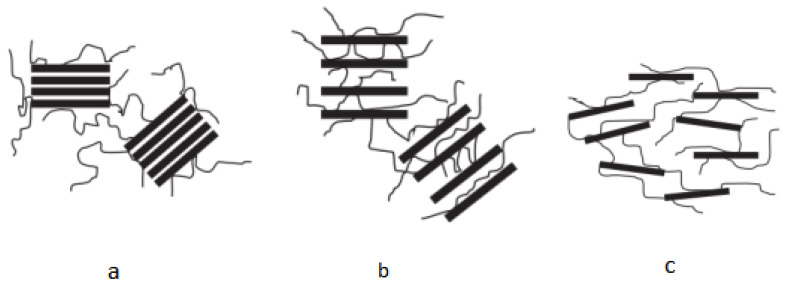
Schematic diagram of various bentonite–polymer nanocomposites: (**a**) phase-separated, (**b**) intercalated, and (**c**) exfoliated structures [43].

The addition of a nano-bentonite composite with a mass fraction of 1% to an aqueous bentonite solution with a mass fraction of 4% results in better filtration performance than organic ammonium salts. In addition, the traditional mud formulation process can be broken and the site simplified if the intercalation complex with bentonite as the primary component and polymer as the second component can be created in the nanoscale as the basic slurry formulation agent for oilfield drilling fluids. The construction assembly approach satisfies the notion of combining convenience and nanocrystallization with a slurry that satisfies the specifications of contemporary drilling fluids [41]. Students from China University of Petroleum used the PAM solution to intercalate bentonite. The findings showed that the smaller the angle corresponding to 2θ in the XRD diffraction pattern and the larger the interlayer spacing, the better the effect of polymer intercalation in preparing nano-bentonite [42]. It is possible to create environmentally responsive polymers that will result in “tunable” polymer-intercalated bentonite nanocomposites. The conformation of responsive polymers varies with external environmental conditions such as pH, ion concentration, temperature, and electric field [43]. In the case of polyacrylamide (PAM), hydrogen bonds occur between the carbonyl oxygen of the PAM molecule and the bentonite surface. Ion–dipole interactions occur between the polar groups of the PAM molecules and the interlayer cations. Three distinct forms of CPNm are produced when bentonite particles bond with polymer molecules in solution: (1) phase-separated (micro-composite or conventional composite), (2) intercalated, and (3) exfoliated structures. In the case of a phase-separated structure (Figure 1a), the bentonite particles are simply dispersed in the system. In the intercalated structure (Figure 1b), the polymer molecules are inserted into the interlayer spaces of the bentonite, but the layers still maintain a well-defined spatial relationship. In the defoliated structure, the layers are completely separated, and the individual bentonite particles are distributed throughout the system (Figure 1c). Nanocomposites were prepared using the solution intercalation technique. The intercalated structure (Figure 1b) is created to maximize the potential for composite modification in the form of interlayer/particle spacing.

The design of organic bentonite materials and their commercial applications depend heavily on a thorough understanding of the intercalation characteristics of organic bentonites. In order to ascertain the bentonite’s interlayer structure morphology, surface composition, and surface functional groups, the study employed X-ray photoelectron spectroscopy, infrared Fourier transform spectroscopy (FT-IR), and X-ray diffraction (XRD) [44]. From the perspective of developing novel bentonite functions, the effect of surfactant type on the interlayer space of montmorillonite is emphasized. Surfactant–bentonite interactions are key to developing new bentonite applications and inorganic–organic nanocomposites. Different concentrations of cetyltrimethylammonium chloride (HDTMA) were used as reference cationic surfactants, polypropylene glycol (PPG) 1200 and 2000 as nonionic surfactants, and Lecithin Topcithin as amphiphilic surfactants modified with phospholipid surfactants. Three intercalation areas were identified based on the surfactant. Nonionic surfactants only cause a crystalline expansion of the bentonite interlayer, whereas cationic surfactants cause osmotic intercalation. Amphiphilic lecithin derivatives are more extensively embedded in bentonite matrices [45]. The process of modifying bentonite with a cationic surfactant (hexadecyltrimethylammonium bromide: CTAB) and silane coupling agent (Hexadecyltrimethoxysilane: HDTMS) is as follows: the intercalation of bentonite by CTAB increases the basal spacing of bentonite, and the thermal stability of bentonite is improved by the covalent bonding of HDTMS with the bentonite layer. The test results showed that the hydrophobicity of the co-modified bentonite was significantly improved and higher than that of the bentonite modified with only a single modifier CTAB or HDTMS. Compared with the bentonite modified by a single modifier, the bentonite modified by CTAB and HDTMS has stable rheological properties and a lower coefficient of friction, which is mainly due to more HDTMS entering the interlayer space of bentonite layers and form chemical bonds with the surface [46].

**Figure 2:**
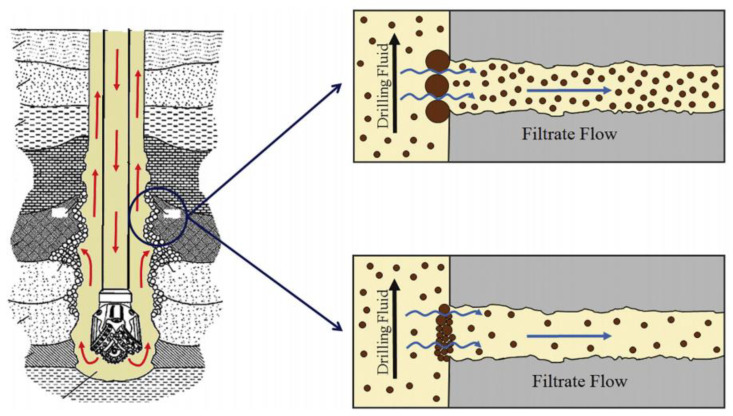
Schematic diagram of the drilling operation and fluid filtration [36].

Drilling fluids are essential for drilling oil, gas, geothermal, and waste disposal wells. Drilling fluids are classified into two types: water-based drilling fluids and oil-based drilling fluids. While oil-based fluids have some advantages in challenging formations, water-based drilling fluids are commonly used in drilling operations for environmental and cost reasons. Water is typically used as the base fluid, together with bentonite and a variety of other additives, such as chemicals and reactive/inert materials, to carry out a number of crucial tasks in the safe and effective drilling of subsurface formations. Drilling fluid is a major factor in most drilling issues, both directly and indirectly, and well-designed drilling-fluid additives can increase drilling efficiency while cutting costs and time [47]. Drilling fluids are used for many different purposes, including suspending drilling cuttings and transporting them to the surface, cooling and lubricating the drill bit, applying pressure to the drilling formation to prevent blowouts, and cleaning the bottom of the hole using a thin, low-permeability filter. The cake wall enhances the well’s strength and lessens fluid loss into adjacent formations, thereby reducing formation damage (Figure 2). Therefore, it must be carefully researched and designed because of its complex physicochemical behavior [48]. A schematic diagram of drilling operations and fluid filtration into adjacent formations due to low- and high-quality filter cake on the borehole wall is depicted in Figure 2. Low-porosity wafers are desirable to reduce fluid losses, prevent pipe sticking, and increase wellbore reinforcement through mud plastering.

During drilling operations, the stability of the wellbore is critical [49]. However, there are other issues as well: the most important one is the hydration of the shale when employing water-based drilling fluids [50]. Amine-terminated polyamidoamine (PAMAM) dendrimers were initially investigated as potential shale stabilizers. Their inhibition properties were characterized by bentonite inhibition tests and shale cuttings dispersion tests. The interactions between different generations of PAMAM dendrimers and sodium bentonite were investigated by XRD, FT-IR, transmission electron microscopy (TEM), and thermogravimetric analysis (TGA). A novel class of water-based drilling fluid with good inhibitory performance has been created, consisting of G0, a shale expansion inhibitor, and G5, a shale dispersion inhibitor. G0 inserts into the interlayer space in a monolayer conformation with increasing concentration, while G1, G2, G3, and G4 insert into the interlayer space in a monolayer arrangement at low concentrations and in a mixed-phase orientation at high concentrations. G5 cannot be fully inserted into the interlayer space because of steric hindrance. According to the interaction between PAMAM and bentonite, the combination of low-generation G0 and high-generation G5 can effectively inhibit the hydration and dispersion of shale [51]. When using water-based drilling fluids (WBDF) for oil and gas drilling, it is imperative to suppress shale hydration in order to maintain wellbore stability [52]. The wellbore instability caused by shale hydration and dispersion has always been a difficult problem in oil and gas drilling engineering [53]. Amino-terminated polyoxypropylene (PEA) is widely used in WBDF to prevent shale expansion and maintain wellbore stability during drilling due to its excellent inhibitory properties and low toxicity to the environment. Understanding the intercalation behavior of PEA between bentonite layers is of great importance for revealing the inhibition mechanism and developing high-performance drilling-fluid additives [54]. The study presented a series of amidosilanols (HAES) modified with methacryloxypropyltrimethoxysilane (MPS) and polyethylene polyamines as potential shale inhibitors to provide resistance in water-based drilling fluids. The development and application of high-temperature shale inhibitors provide conditions. X-ray diffraction, adsorption measurement, thermogravimetric analysis, and transmission electron microscope analysis were used to examine the corrosion inhibition mechanism of HAS-8. The mechanism is the surface adsorption and interlayer intercalation of bentonite, which inhibits the surface hydration and osmotic hydration of bentonite. In the field application of Well Bei 9, HAS-8 effectively suppressed the hydration of water-sensitive cuttings, ensured the wellbore stability of the shale formation, and provided a guarantee of safe and rapid drilling [55].

The direct intercalation of three green ionic liquids (ILs) with varying salt and cation sizes into bentonite interlayers was studied. The ionic liquids involved in this study include 1-hexyl-3-methylimidazolium chloride (IL-1), 1-butyl-3-methylimidazolium octyl sulfate (IL-2), and 1-butyl- 3-Methylimidazolium bromide (IL-3). The IL modification of bentonite aims to improve the rheology and thermal stability. This goal is achieved by increasing the expansion of the bentonite interlayer. The fundamental spacing of the ILs intercalated bentonite and the dry bentonite powder detected by XRD showed that the cations of the three ILs were successfully intercalated into the interlayer of the bentonite layers through a cation exchange mechanism, and the swelling might be influenced by the size of the cations, type, and concentration [56].

### 3.2. The Method of Melt Intercalation

Polymer melt intercalation is the direct insertion of a polymer above its softening point between bentonite silicate layers under static or shear forces. With its broad variety of applications and promising future applications, the polymer melt intercalation technology eliminates the need for solvents, simplifies the production process, and reduces environmental pollution [57,58].

In addition to being reliant on the interaction of the polymer matrix with the silicate layer intercalation agent, mixing shear conditions has a significant impact on the degree of dispersive exfoliation by melt intercalation. Factors such as melt viscosity, shear rate, shear strength of the extruder to the melt (e.g., screw configuration), mixing temperature, and residence time all influence the effect of intercalation and the final properties of the composite [27]. Since Vaia et al. initially showed that direct melt mixing and intercalation are used to generate polymer/Mt nanocomposites, the technique is quite near to being produced industrially, requires nothing in the way of processing equipment, and does not require the use of solvent advantages [59,60]. Melt intercalation has become the main method for preparing polymer/Mt nanocomposites. Pengcheng Ma [61] prepared polylactic acid (PLA)/montmorillonite (Mt) nanocomposites by melt intercalation and characterized their properties. Research showed that organic modification of Mt can increase the interlayer spacing of Mt. In addition to enabling the formation of exfoliated and intercalated structures, this can significantly enhance the qualities of PLA/Mt composites. By observing the changes in surfactant coverage when polystyrene (PS) is melted and intercalated into organic bentonites, it can be seen that the surface coverage of the organic bentonites plays an important role in controlling the type of final composite formation. Two main types of composites were found based on the surface treatment of organic bentonites. These consist of conventional composites and intercalated nanocomposites. Conventional composites were formed in cases where the bentonite surface coverage was higher. It is characterized by micron-sized aggregates of organic bentonite particles. In the organic bentonites with low surface coverage, intercalated nanocomposites were observed. As the polystyrene intercalates into the organic bentonite interlayer, the organic bentonite is dispersed into a smaller pile. This is because of the affinity between the organic bentonite surface and molten polystyrene [62].

**Figure 3:**
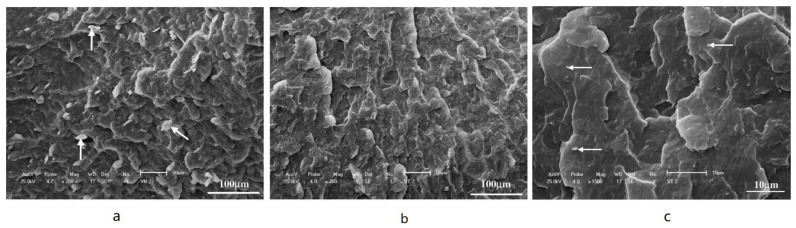
SEM images of compounds containing 2 wt% bentonite. (**a**) PP/PB; (**b**) PP/Mt (the arrows indicate the filler agglomerates); (**c**) PP/Mt shown in (**b**) under higher magnification. The arrows indicate the presence of bentonite particles [64].

Melt intercalation is a very successful method for producing polymer–bentonite nanocomposites. Techniques including thermogravimetric analysis (TGA), scanning electron microscopy (SEM), and X-ray diffraction (XRD) are typically used to characterize the produced materials. Experiments determine the amount of polyethylene oxide (PEO) required to saturate the interlayer spacing of montmorillonite (Mt) and organically modified bentonite (B34). Differential scanning calorimetry (DSC), XRD, and TGA are used to calculate the saturation ratio of PEO to silicate and compared with theoretical calculations. By XRD and DSC, the saturation ratio of PEO and montmorillonite is 28:72, and the saturation ratio of PEO and B34 is 15:85. By TGA, the saturation ratio of PEO and montmorillonite is 21:79, and the saturation ratio of PEO and B34 is 21:79. The density of intercalated PEO in the silicate corridor is estimated to be 0.82 g/cm^3^, indicating that the filling efficiency of PEO in the silicate corridor is much lower than that of the bulk polymer in the amorphous region [63]. XRD and Fourier transform infrared (FT-IR) studies were used to compare polyethylene oxide/sodium montmorillonite (PEO/Mt) and PEO/organic alterations made by solution intercalation and melt intercalation. In the PEOMt and PEO/B34 systems, the size of the solution intercalated hybrid material grew with the increase in PEO content and reached a stable level of 15%. Regardless of the PEO concentration, the gallery size of the melt-intercalated PEO/Mt and PEO/B34 hybrid materials did not change. FT-IR analysis showed that there was no spectral difference between the samples prepared by solution intercalation and those prepared by melt intercalation [51]. Polycationic bentonite (PB) was organically modified with quaternary ammonium salts and added to isotactic polypropylene (PP). The compounds were prepared by melt intercalation using a twin extruder and then characterized by XRD, TGA, and SEM to clarify the composite nanostructures. The carbonyl index results obtained by infrared spectroscopy showed that the modified bentonites have higher thermal stability in the solid state compared to the natural bentonites. This is related to the higher dispersion of the bentonite particles. On the one hand, the degradation of the composite was faster than that of the unfilled polymer. This is because there are acidic sites on the bentonite’s surface, which act as catalysts for polymer oxidation. In addition, salt decomposition initiates the radical degradation of PP. The dispersion of bentonite in polypropylene after liquid nitrogen fracture was observed using a scanning electron microscope. Figure 3a,b show that the unmodified bentonite (PB) has uneven dispersion, large agglomerates, and poor adhesion to polymers. On the other hand, no agglomerates were observed in the modified bentonite at the same magnification. There are some bentonite particles visible in the matrix structure at higher magnification (Figure 3c). In unmodified and modified bentonite, the particle agglomeration sizes were 20 mm and 1 mm, respectively. The presence of small aggregates in the modified bentonite is consistent with X-ray analysis, which indicated that both nanocomposites and micro-composites were formed [64].

The effectiveness of melt intercalation can be further improved by using microwave (MW) irradiation. The energy is mainly absorbed by the water molecules coordinated with the interlayer cations. The MW-assisted melt intercalation process is successfully applied for the first time to the preparation of montmorillonite–PEO nanocomposites, saving time and energy compared to the conventional method of oven-heating.

Alkyl aluminum compounds are used to chemically modify sodium bentonite in order to create polypropylene nanocomposites that contain uniformly shaped nano-bentonites. This compound was compared to PP/commercial organic bentonite containing alkylammonium ions. Both unmodified and modified bentonite compounds were incorporated into commercial PP matrices and mixed with maleic anhydride PP (PP-MA) by melt intercalation using a small extruder. The resulting bentonite/polymer nanocomposites (CPNs) were characterized by differential scanning calorimetry, X-ray diffraction, thermodynamic-mechanical analysis, and thermogravimetric analysis. According to the results of the method developed in this study, the prepared PP nanocomposite bentonite showed an increase in the d-value of the bentonites in the XRD and resulted in better properties such as degradation temperature and increased mechanical parameters. And the treatment with triethyl aluminum worked best [65].

## 4. Grafting Modified Bentonite

The most commonly used method for modifying organic bentonite for drilling fluids is intercalation. It is difficult for polymer intercalated bentonite to proceed spontaneously because it requires an internal or external driving force. With the widespread use of nanotechnology, the method of surface grafting has gradually been applied. Nanoparticle surface modification methods include chemical modification and physical modification. The principle of physical modification is as follows: the modifier is adsorbed on the surface of nanomaterial particles and covers the hydroxyl groups on the surface of nanomaterials; it leads to the proportion of hydroxyl groups on the surface of nanomaterials changes and the effect of surface modification of nanomaterials are obtained. The principle of chemical modification is as follows: the modifier reacts with the hydroxyl group on the surface of the nanomaterial or the introduced unsaturated bond; this grafts the surface of the nanomaterial with long chains, changing the surface structure and state and achieving the effect of surface modification. The chemical modification methods mainly include the coupling agent modification method and surface graft polymerization method.

### 4.1. The Method of Physical Grafting

Physical modification mainly achieves the effect of particle surface modification through physical adsorption. The substance is deposited on the surface of nanoparticles to form a heterogeneous coating layer that is not chemically combined with the surface of the particles to achieve the purpose of inhibiting the aggregation of nanoparticles. Scholars have used this method to deposit silver on the surface of nano-silica and achieved a good modification effect. The agglomeration of nanoparticles can be prevented by adsorbing heterogeneous materials on the surface of nanoparticles through van der Waals forces, etc. [66]. By studying the adsorption behavior of various types of polyacrylamide on the surface of nano-SiO_2_, Samoshina et al. found that the addition of the surfactant Tween 80 can significantly enhance the adsorption effect of polyacrylamide on the particle surface [67]. Lin et al. treated nanoparticles with polyacrylic acid, effectively improving the coagulation phenomenon of nanoparticles and stabilizing the dispersion of nanoparticles in the aqueous phase [68]. Li Xinjun et al. used stearic acid to modify nano-CaCO_3_ to improve the dispersion of nano-CaCO_3_ [69]. The dispersion stability of nano-CaCO_3_ in water was improved by the surfactant sodium polyacrylate, and the influence of pH on the effect of the dispersant was analyzed. The experimental results show that as the pH value increases, the dispersion stability of nano-CaCO_3_ in the system is enhanced [70].

The physical modification process is simple and easy to operate, but this method is adsorbed on the particle surface by van der Waals forces, hydrogen bonds, and other weak forces. Under external stimuli, such as high-speed stirring, high-temperature heating, etc., the forces between the surfactant and nanometer are easily destroyed, so the nanomaterials modified by this method are relatively unstable.

### 4.2. The Method of Chemical Grafting

Chemically initiated grafting is a reaction between chemical reagents and polymer surface components to create active sites and initiate the polymerization of monomers. For example, reacting a monomer containing an azo group with a hydroxyl group on the surface of the polymer and introducing it into the surface of the polymer can initiate the polymerization of the monomer on the surface of the polymer by thermal decomposition of the azo group.

#### 4.2.1. The Method of Coupling Grafting

Coupling grafting means that the reaction between the reactive group of the grafted polymer and the group on the grafted polymer can be realized by coupling a certain substance. Silane coupling agents are most effective for inorganic nanoparticles with hydroxyl groups on the surface. Their combination with nanoparticles begins with the interaction of silane oligomers with hydroxyl groups on the surface of nanoparticles. Therefore, inorganic materials with active hydroxyl groups on the surface, such as glass, silica, etc., have strong affinity and reactivity with silane coupling agents. In contrast, the treatment effect is poorer for inorganic materials with no hydroxyl groups or low polarity on their surface, such as calcium carbonate, carbon black, etc. [71].

**Figure 4:**
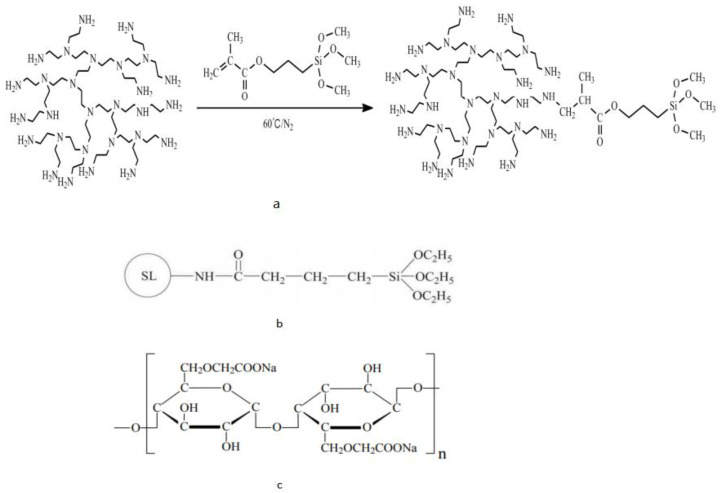
Molecular structure diagram: (**a**) schematic diagram of the synthesis of Si-HPEI; (**b**) molecular structure of Si–SL; (**c**) structural formula of carboxymethyl cellulose [72,73,74].

Silane coupling agent (Si-HPEI) modified hyperbranched polyethyleneimine shale was synthesized by Michael’s addition reaction of hyperbranched polyethyleneimine (HPEI) with 3-Methacryloxypropyltrimethoxysilane inhibitor. Figure 4a shows its synthesis process. The results show that, compared with HPEI and traditional shale inhibitors, Si-HPEI can effectively inhibit the hydration expansion of shale and has excellent temperature resistance. Mechanistic analysis shows that the siloxane groups in the molecular chain of Si-HPEI lead to strong and stable chemisorption of Si-HPEI on water-sensitive bentonite, thereby hindering the penetration of water molecules and finally inhibiting the water-sensitive bentonite minerals of water-sensitive bentonite minerals, chemical expansion, and dispersion. In the field application, Si-HPEI was successfully applied on the Su 4-4HF well [72].

The challenges associated with drilling operations are numerous, the adverse effects of which can result in severe damage to or even the shutdown of drilling operations. The most serious problem drilling engineers encounter when working with water-based drilling fluids (WBDFs) is wellbore instability. This is because shale formations are rich in reactive bentonite minerals, which can cause hydration and swelling when in contact with WBDF and damage the integrity of the wellbore [75]. Some of the silane coupling agent molecules can react with various groups on the surface of the montmorillonite crystal; some of them called the modifier can chemically react with or adsorb the organic matter. The use of silane coupling agent to modify the montmorillonite crystal organic modifier could strengthen the combination of organic modifier and montmorillonite crystal surface and make it more stable [76]. Silane coupling agent (Si–SL)-modified sulfonated lignin (SL) was investigated as a biodegradable shale inhibitor for water-based drilling fluids (WBDF). (The molecular structure of Si–SL is shown in Figure 4b). The results show that the corrosion inhibition performance of a series of Si–SL is similar to that of Ultrahib and significantly better than HCOOK, PEA, and K-PAM at room temperature. Furthermore, Si–SL exhibits the best temperature resistance compared to other inhibitors. At high temperatures, Si–SL can be strongly adsorbed on the surface of bentonite particles, and the phenyl group in the Si–SL molecular chain is beneficial to improve the temperature resistance and hydrophilicity of the bentonite particles’ surface, thereby effectively preventing the migration of water molecules to the bentonite layer. Moreover, through the biotoxicity and biodegradability tests, the results show that Si–SL has no toxicity and easily biodegrades. In the field application of Bei 213-21HF well, Si–SL effectively inhibited the hydration of water-sensitive cuttings, maintained the wellbore stability of the shale formation, and provided a guarantee of safe and rapid drilling [73]. The effects of different silane coupling agents on the mechanical strength, tribological properties, thermal properties, swelling properties, and rheological properties of bentonite (Bent)/nitrile butadiene rubber (NBR) nanocomposites were investigated. The results show that different functional groups of silanes are effective on the properties of nanocomposites, mainly due to their different interactions with the rubber matrix [77].

Silane coupling agent KH570 and modified nano-silica with natural rapeseed oil were used to prepare a loss circulation material SD-NR. Adding 2% of this loss circulation material reduces the friction coefficient from 0.43 to approximately 0.04. Moreover, the extreme pressure film strength formed by the drilling fluid with this lubricant reaches about 250 MPa, withstanding over 180% of high-temperature conditions. It is a lubricant with excellent performance [78]. A polymer skeleton matrix with certain high-temperature resistance was prepared by using low-viscosity PAC, styrene, acrylic acid, acrylamide, 2-acrylamido-2-methylpropanesulfonic acid, etc., as raw materials. The target fluid-loss control agent Psi-1 was prepared by adding SiO_2_ nanomaterials as fillers and silane coupling agents as binders. Orthogonal experiments were designed with the conditions of initiator dosage, reactant ratio, and reaction temperature as independent variables to test the performance of the product. The product was added to a 4% base slurry, and its fluid-loss performance, rheological performance, high-temperature resistance, lubricity, etc., were tested. Graphene material was added to this simple system, the influence of its dosage on the drilling-fluid system was analyzed, and the fluid loss was enhanced by reducing the ability of the fluid-loss reducer through the compound use of nanomaterials [79].

Due to the presence of a large number of active hydroxyl groups on the surface of nano-SiO_2_, the surface of nano-SiO_2_ has hydrophilic and oleophobic properties, which makes it easy to agglomerate and is difficult to disperse in organic material or polymers. It is difficult to form coupling bonds between fillers and polymers, thus limiting the role of nano-SiO_2_. Therefore, researchers at home and abroad have modified nano-SiO_2_, that is, grafting hydrophobic groups on the surface of nano-SiO_2_, reducing the number of hydroxyl groups on the surface, changing it from hydrophilic and oleophobic to hydrophobic and lipophilic, enhancing the interaction of nano-SiO_2_ with compatibility between organic materials [80]. Modified nano-SiO_2_ with γ-methacryloyloxytrimethoxysilane KH570, a coupling agent with unsaturated double bonds, introduced double bonds into the surface of nano-SiO_2_, and alkylated it, which greatly improves dispersion stability [81]. Using a silane coupling agent to hydrophobically modify nano-SiO_2_, a nano-plugging material was developed. The blockage rate increased from 71% to 88% [82]. In the nano-SiO_2_ modification experiment, vinylsilane and aminosilane were selected as surface modifiers acting on the surface of the particles, and the modification effects of the two coupling agents were analyzed through characterization and performance evaluation experiments. The experiment found that the hydrophobicity of the nano-SiO_2_ modified by the two modifiers was significantly improved, and the dispersion stability of the nanomaterials in the system was significantly enhanced [83]. Nanometer-sized TiO_2_ particles were modified with the silane coupling agent WD-70, and the nanomaterials before and after modification were characterized and evaluated. Experiments show that surface modification of the silane coupling agent WD-70 significantly improves the dispersion of nano-TiO_2_ [84].

Nanoparticles are treated with vinyl-containing silane coupling agent, and vinyl groups are introduced on the surface of Co_3_O_4_ particles. Subsequently, using K_2_S_2_O_8_ as an initiator and sodium dodecylbenzenesulfonic (SDBS) as an emulsifier, emulsion polymerization is employed to graft methyl methacrylate onto the surface of Co_3_O_4_ particles [33]. The surface of nano-CaCO_3_ was modified with water as a dispersion medium and silane coupling agent as a modifier. The experimental results showed that the lipophilicity of nanomaterials was improved after modification [85]. Tri alkoxysilane coupling agents were used to modify the surface of nano-CaCO_3_ particles, and polymer-polyethylene was grafted onto the surface of particles, finding the dispersion to be more stable [86]. A stable molecular film formed on the surface of the particles after a chemical reaction between the titanate coupling agent and the free protons of nano-calcium carbonate [87]. Compared with unmodified CaCO_3_, filling PVC with nano-CaCO_3_ modified by aluminate coupling agent can significantly improve its fracture elongation and impact strength [88]. The result showed that the oil absorption value and water absorption rate were reduced, and the dispersibility was good. The silane coupling agent modification method has a simple experimental operation, easy-to-satisfy experimental conditions, and good repeatability of the testing effect. It is the most effective and widely used nanomaterial modification method at present [89].

#### 4.2.2. The Method of Graft Copolymerization

The graft polymerization method refers to a process in which organic monomers are polymerized on the surface of nanoparticles or a polymer is grafted onto the surface of nanoparticles through a polymer reaction. The polymers directly adsorb on the surface of inorganic nanoparticles, or aggregate and bind on the surface of the particles, causing the nanoparticles to repel each other and disperse stably in the system.

Carboxymethyl cellulose is a water-soluble cellulose ether compound [90]. Its structure is shown in Figure 4c. The preparation process of carboxymethyl cellulose is simple, and it is easy to obtain the raw materials. The most attractive is its excellent water solubility, which makes it easy to perform graft polymerization in aqueous solution and ensures the uniformity of the reaction. Graft polymerization overcomes the heterogeneous reaction with other cellulose. At the same time, carboxymethylcellulose graft copolymerization is an important chemical method in polymer modification technology. The research on the graft copolymerization of hexomethylcellulose has broad development prospects and practical significance. The results of the successfully prepared carboxymethylcellulose-grafted acrylamide copolymers show that the concentration, salt resistance, and heat resistance of the graft copolymers in the aqueous solution are greatly improved [74]. CMC-AM graft copolymer is prepared by graft modification with acrylamide (AM), with ammonium sulfate and sodium bisulfite as the initiator and carboxymethyl cellulose (CMC) as the main raw material. The addition of AM/CMC graft copolymer significantly enhances the performance of drilling fluids across various systems including freshwater drilling fluid, 4% saltwater drilling fluid, saturated saltwater drilling fluid, and calcium-containing saltwater drilling fluid. It significantly reduces the filtration rate while demonstrating improved rheological properties, indicating excellent salt resistance of the graft copolymer within the drilling fluids. The AM/CMC graft copolymer drilling-fluid treatment agent has anti-temperature and anti-aging properties in different systems of drilling fluids. The measurement results show the good rheological properties and fluid-loss properties after aging, indicating that the AM/CMC graft copolymer has good temperature resistance and aging resistance in the drilling-fluid system [90].

The carboxymethyl grafted polyacrylamide copolymer was synthesized by free-radical polymerization technology. The study showed that the synthesized graft copolymer had a significant effect on the rheology and filtration performance of the corrosion-inhibited drilling-fluid system, and better performance in shale recovery. Therefore, inhibited drilling-fluid systems with synthetic graft copolymers can be used for drilling water-sensitive shale formations [91]. A novel graft copolymer (CSGO) was synthesized by radical polymerization using carboxymethyl chitosan (CMCS). The potential application of CSGO in sodium bentonite (Na Bent) dispersions was investigated through rheology, filtration, and inhibition performance tests. The results show that CSGO improved the rheology and filtration performance of Na Bent dispersions before and after aging at 120 °C due to the combined results of the aggregation and film formation of bentonite particles [92].

Shale formations composed of montmorillonite-like active bentonite minerals cause serious wellbore instability problems with conventional water-based drilling fluids. The applicability of the synthesized polyacrylamide/dimethylammonium chloride grafted Arabic gum copolymer in the formulation of a novel water-based drilling mud system (WBDMS) in complex shale formations was discussed. The mechanism of synthesis is free-radical polymerization. Experimental studies have shown that the synthesized graft copolymers have a synergistic effect in the developed system and have a significant effect on the rheological parameters and filtration properties of the system [91]. In order to solve wellbore instability during the drilling process, it is critical to use high-performance shale stabilizers in water-based drilling fluids.

A novel shale stabilizer based on polyethylene glycol-grafted nano-silica composites (PEG-NSs) was successfully synthesized. The experimental data show that PEG-NSs can effectively delay the transmission of pore pressure and reduce the permeability of shale samples. PEG-NSs are adsorbed on the shale surface and eventually covered with a dense blocking film [93].

In recent years, with the gradual deepening of oil exploration and development to deep levels, domestic and foreign scholars have begun to focus on the research of environmentally friendly high-temperature-resistant modified starch for drilling fluids. Temperature-resistant modified starch is mainly divided into two directions. The graft copolymerization modified starch has better temperature resistance but is not easy to degrade. Although the traditional etherified modified starch has environmental protection characteristics, its temperature resistance is poor, and the temperature resistance is generally below 130 °C. So, in-depth research on environmentally friendly and high-temperature-resistant modified starch with a resistance temperature above 130 °C is required [94]. Since starch graft copolymers have both the salt resistance of starch and the temperature resistance of polymer products, which meets the needs of the development of drilling-fluid treatment agents, the research on starch graft copolymers for drilling fluids has gradually increased. However, these studies rarely achieve industrial production in the present, and the focus of future work should be on product industrialization and on-site application [95]. Using starch as raw materials, various oilfield chemicals for different purposes can be obtained through graft copolymerization with monomers like acrylamide, acrylic acid, acrylonitrile, 2-acrylamido-2-methylpropanesulfonic acid, 3-methacrylamidopropyltrimethylammonium chloride, diallyldimethylammonium chloride, and others. The research and application results show that the starch graft copolymer is used as a drilling-fluid treatment agent and has strong filtration reduction ability and good salt and temperature resistance in freshwater, brine, saturated brine, and composite brine drilling fluids. The starch graft copolymer is used as a profile control and water shutoff agent; it can be cross-linked with many cross-linking agents to form a highly viscoelastic gel system. The flexible side chains and the rigid skeleton in the molecular structure penetrate each other to form a star-shaped or comb-shaped structured polymer. And its cross-linking system has strong temperature resistance, salt resistance, shear resistance, and long-term stability. Starch graft copolymers are used as flocculation and purification agents for oilfield water treatment due to their abundant sources, low prices, and high availability. They have the characteristics of biodegradation, strong flocculation, good coagulation, and decolorization effect [96].

Scholars from the South China University of Technology polymerized methyladienoic acid (MAA) to form polymethacrylic acid (PMAA) polymer, which was grafted onto the TiO_2_ surface to improve its dispersion in water. The structure was characterized by infrared spectroscopy and thermal analysis, and the results showed that the polymethyladienoic acid was successfully grafted onto the surface. However, during particle size analysis, the relatively pure particle size of the TiO_2_ grafted sample increased. After TiO_2_ is grafted with PMAA with polymer chains, the movement of the particles in the water becomes slower, and the particle size measured on the instrument increases. It is unscientific to rely solely on particle size analysis for characterization of grafted polymer nanoparticles the dispersion effect. Therefore, it should be comprehensively considered in combination with the absorbance curve of the dispersion solution [97]. The nanoparticles are treated with a silane coupling agent and functionalized by introducing double bonds to their surfaces. The surface of SiO_2_ nanoparticles is grafted and polymerized through solution polymerization to achieve the surface coating treatment of the polymer on SiO_2_ nanoparticles, thereby preparing the composite materials. And the surface structure of nanoparticles is characterized by IR and XPS [98]. In addition, some researchers grafted hyperbranched polymers or hydrophobically associative polymers onto the surface of nano-SiO_2_ to form a nanocomposite with a core–shell structure, improving the dispersibility and compatibility of nano-SiO_2_. Due to the special branching structure and hydrophobicity of hyperbranched polymers, they can penetrate shale pore throats or fractures for plugging [99,100].

In order to improve the high temperature, salt, and calcium resistance of water-based drilling fluids in deep and ultra-deep drilling exploration, poly (anionic cellulose), 2-acrylamide-2-methylpropanesulfonic acid (AMPS), diallyldimethylammonium chloride (DMDAAC), N,N-dimethyl Acrylamide (DMAA), using sodium p-styrene sulfonate (SSS) and nano-silica as a raw material, were used to form copolymer PAC-DDAS-SiO_2_ by free-radical copolymerization in aqueous solution. The monomers were polymerized using X-ray photoelectron spectroscopy (XPS). After adding the copolymer to the drilling fluid, the rheological properties of the drilling fluid were maintained. The article analyzed the filtration volume after aging at 260 °C, the microstructure of bentonite polymer interaction in the drilling fluid, and the particle size of the drilling fluid. There are numerous anionic groups on the ball chain of the copolymer, which form hydrogen bonds and ionic bonds with the montmorillonite to ensure the normal size (less than 2 microns) of the bentonite particles, resulting in a wider particle size distribution phenomenon in the drilling fluid, dense filter cake, and reduced filter volume [101]. Using humic acid as the skeleton, sulfobenzaldehyde (SMP) resin was grafted into the water–bentonite slurry by water polycondensation to synthesize lignite-grafted branch polycondensate with water retention and dispersion properties. The characterization results of the chemical structure and molecular weight of the polycondensate showed that the grafting of SMP was successful. The obtained lignite-grafted polycondensate was used as a drilling-fluid additive, effectively controlling the filtration volume and exhibiting rheological stability at aging temperatures up to 200 °C. The behavior of polycondensates differentiates them from most common synthetic high-temperature fluid-loss reducers such as SMPs, which tend to excessively thicken the slurry. The colloidal properties of water–bentonite mixtures were studied through adsorption and zeta potential experiments [102].

The surface graft polymerization method can not only effectively improve the dispersibility of nanomaterials but also introduce new functional groups to the surface of the particles to endow nanomaterials with new functions [32]. Research has found that 3-vinyltriethoxysilane (MSP) was first modified, then styrene polymerization was initiated with an azodiisobutyronitrile initiator, and graft copolymerization was carried out with a modifier. The thickness of the grafted layer and the kinetics of the grafting process were studied, and it was found that the graft thickness was as high as 15 nm, and the dosage of the initiator and monomer concentration did not affect the grafting thickness. Chemical treatment methods are more effective in promoting mixing between nanoparticles and polymers, utilizing monomer permeation during polymerization to facilitate the dispersion of agglomerates. Cetyltrimethylammonium bromide (CTAB)-grafted polyacrylamide (PAM) gel for fluid-loss control in high-performance drilling fluids with improved thermal stability was used [103]. Using green chemistry techniques, PAM was treated with CTAB to prepare PAM complexes. The addition of the PAM complex improves the rheology of the mud system and increases the fluid retention capacity [104].

#### 4.2.3. Other Chemical Reaction Methods

In the field of modified organic bentonite, a variety of modified products have been synthesized through polymer chemical reactions such as esterification, etherification, sulfonation, complexation, and cross-linking [105]. At present, the commonly used etherified modified starch fluid-loss reducer in oilfields is carboxymethyl starch (CMS). Its heat resistance can be enhanced with an increase in substitution degree, while its anti-corruption ability is also improved. Therefore, new etherified modified starch with a high degree of substitution should be developed and produced, so that its single-agent temperature resistance can reach 120~130 °C or above. The application in oilfield drilling will be one of the development directions of high-temperature-resistant modified starch. Students at Changjiang University have developed a composite ionic-modified starch fluid-loss reducer CSJ for indoor use. It is firstly replaced by anions and then by cations. It is required to control the degree of substitution in each reaction stage to ultimately form a temperature modified starch fluid-loss reducer, which has a good water loss reduction effect in 3% bentonite slurry. The temperature resistance of the system can reach 140 °C, but the temperature resistance and salt resistance performance of this single agent has not been reported [106]. Modified corn starch drilling-fluid-loss reducer was synthesized indoors. This is a type of temperature-resistant modified starch obtained by silicification first and then etherification. The temperature resistance of a single agent can be up to 110 °C. When the concentration is 2%, the temperature resistance of the modified starch in the mud can be up to 130 °C, and it has a good effect in reducing filtration loss [107]. Two types of starch, grafting thermal cross-linking and etherification, were synthesized using potato starch as a raw material. These two types of starch can be used alone or in combination as loss reducers and can partially or completely replace CMC-type drilling-fluid-loss reducers in the future [108]. Corn starch (CS) is used as raw material, chloroacetic acid is used as the first-step etherification denaturant, and sodium 3-chloro-2-hydroxypropyl sulfonate is used as the second-step etherification denaturant to synthesize a compound etherified starch (CES) fluid-loss reducer that can be used in oilfield drilling fluids under alkaline conditions. The results of the temperature resistance test showed that the anti-aging temperature of the compound etherified carboxymethyl sulfonate starch prepared by the optimized process conditions can reach 130~140 °C and has relatively good filtration loss reduction performance. It can be used as a filtrate reducer in drilling fluid [109]. Using corn starch, ClCH_2_COOH (MCA), and epichlorohydrin CH_20_CHCH_2_Cl (ECH) as raw materials, a filtration control agent, CCMS, was produced through etherification, cross-linking, and composite modification. The product exhibits excellent filtration control properties in freshwater, 4% saltwater, saturated saltwater drilling fluids, and freshwater drilling fluids containing 40% CACl_2_, maintaining its effectiveness at temperatures up to 160 °C [110]. To enhance the high-temperature resistance of starch-based filtration control agents, researchers have modified starch with inorganic silicon, synthesizing starch-based filtration control agents with excellent high-temperature resistance [111]. Additionally, utilizing organic silicon (hexamethyldisilazane) for further modification of etherified starch has led to the preparation of organic silicon starch-based filtration control agents resistant to high temperatures [112].

The polyacrylate intercalated bentonite superabsorbent hybrid material was prepared by the solution polymerization method through intercalation, polymerization, and cross-linking reactions. Intercalate partially neutralized acrylic acid (NaA) into bentonite for polymerization and cross-linking. The initiator is potassium persulfate, and the cross-linker is sugar. The structure of the polyacrylate/hybrid was determined by FT-IR and XRD. The results indicate that the interlayer spacing of polyacrylate intercalated bentonite increases. The effects of neutralization degree, monomer dosage, initiator and cross-linking agent dosage, bentonite dosage, and other factors on the water absorption of resin were studied through orthogonal experiment. The optimal conditions are that the mass ratio of bentonite to monomer is 1/2, the degree of neutralization is 75% (mol), the content of initiator is 3%, and the content of cross-linked agent is 5%. The cost of superabsorbent resin is reduced by 30%, while its water absorption is 120 g/g for water and 30–36 g/g for brine [113].

Wellbore instability is related to shale expansion as it interacts with free water molecules in water-based drilling fluids. The strategic design of environmentally friendly, biodegradable, and effective shale hydration inhibitors is a prominent goal of modern drilling-fluid exploration to replace the conventional substance KCl, which is harmful to aquatic organisms. To explore the synthesis of a novel green acrylic polymer amyl ester activated carbon (-C) nanocomposite and its potential to prevent shale hydration in formations during drilling, the less hydrophobic acrylamide-activated carbon amyl acrylate (AA-AAm-C-amyl) and the stronger hydrophobic acrylamide octadecene-activated carbon amyl (AA-AAm-OD-CAmyl) composites were synthesized. They were characterized and tested by standard methods as a cleaning fluid additive to inhibit shale expansion, and the results were compared to KCl. The polymer matrix exhibits significant thermal stability. The results also show that the AA-AAm-C-pentyl and AA-AAm-OD-CAmyl composites can effectively stabilize the wellbore with a stabilization rate of 95% and a shale recovery capacity of 97% [114]. Early research on the esterification reaction of fentanyl succinic anhydride in weakly alkaline water with starch, acetic acid (anhydride), or propionic acid (anhydride) also showed hydrophobic properties after the reaction with starch [115,116].

## 5. Molecular Simulation of Organic Bentonite Modification

**Figure 5:**
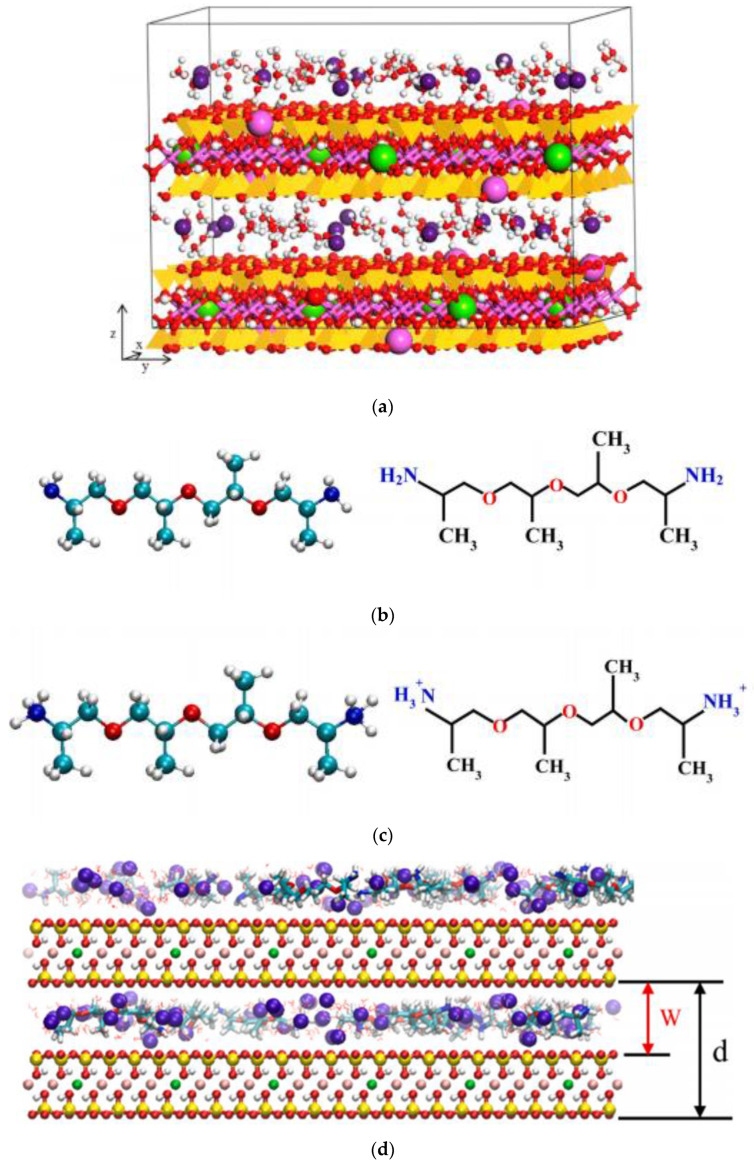

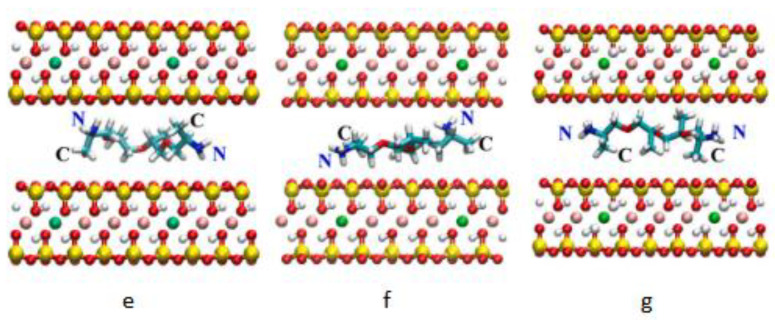
(**a**) Snapshot of one of the simulated Cs—montmorillonite systems with water content corresponding to 6 H_2_O molecules per Cs^+^ ion or 5 H_2_O per unit cell (1-layer hydrate) [119]; molecular structure and chemical formula of (**b**) neutral PEA and (**c**) protonated PEA. Color scheme: blue—N; red—O; cyan—C; white—H. (**d**) Snapshot of equilibrated systems containing neutral PEA molecules with the water content of 0.1 g/g bentonite. Color scheme: violet—Na; blue—N; red—O; cyan—C. Snapshot of PEA molecules in scenario (**e**) Scenario I, (**f**) Scenario II, and (**g**) Scenario III at the condition of one layer water content. The water and ions are removed for clarity. (**a**) Scenario I-1 W, (**b**) Scenario II-1 W, and (**c**) Scenario III-1 W [54].

The study of chemical processes and material development at the molecular level is an important frontier in chemistry and chemical engineering. The emergence of many new chemical technologies is accompanied by some complex microscopic processes, which require a deep understanding of the mechanisms of various complex phenomena. Therefore, it is necessary to study the microstructure of the system at the molecular level and establish the relationship between the microstructure and the macroscopic properties. Molecular simulation is considered one of the key technologies for achieving this goal [117,118]. Many molecular simulations have been reported to study the swelling behavior and interlayer structure of bentonite (Figure 5a) [119]. In the experiments with the polyether amine intercalated sodium-based montmorillonite swelling inhibitor, all simulations were performed using the LAMMPS software to obtain the interfacial thermodynamic and structural properties. Figure 5b,c show the molecular structures of neutral PEA (N-PEA) and protonated PEA (P-PEA) molecules. The equilibrium configuration of the system containing neutral PEA molecules is shown in Figure 5d. To better explain the distribution of N and C atoms under different conditions, a representative snapshot of the PEA configuration is shown in Figure 5e–g [54].

Many scholars have revised and improved Vaia’s mean-field theory, simulated the intercalation process of layered silicates with polymer macromolecules or solvent small molecules [120], providing a better basis for the theoretical analysis of the intercalation process. The data from the same process were calculated using molecular dynamics simulation. Based on the simulation results, the free energy of the system can be roughly estimated, and the value of the interlayer spacing value of layered silicate after reaching a stable state in an aqueous medium can be estimated. According to these calculation methods, the thermodynamic state of the macromolecules intercalated into the layered silicate can be further calculated, and various parameters of the system in the stable state can be estimated [121]. The research group represented by E.P.Giannelis has achieved considerable results in this field. With the advancement of experimental testing methods and computer simulation technology, this theory continues to be improved and developed. Using the Monte Carlo molecular simulation method and based on the principle of minimum energy, the swelling properties and interlayer structure of sodium montmorillonite and surfactant-modified organic montmorillonite in a carbon dioxide environment were studied at the microscopic level. The simulation results show that the interlayer carbon dioxide has an obvious multi-layer distribution phenomenon with the increase in the interplanar spacing of montmorillonite, and the interlayer surfactant forms different structural forms with the change in carbon dioxide content [122,123].

**Figure 6:**
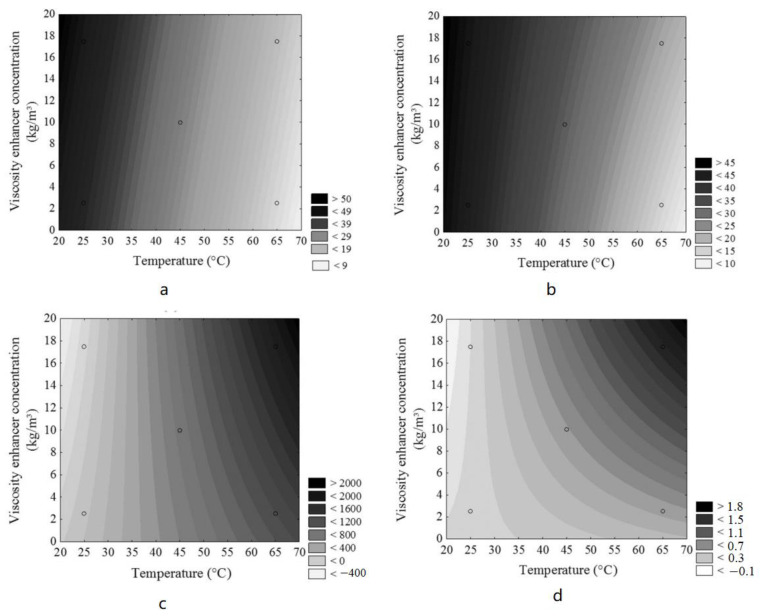
Surface plot (fitted response) to (**a**) plastic viscosity, (**b**) apparent viscosity, (**c**) thixotropy, and (**d**) yield strength [124].

**Figure 7:**
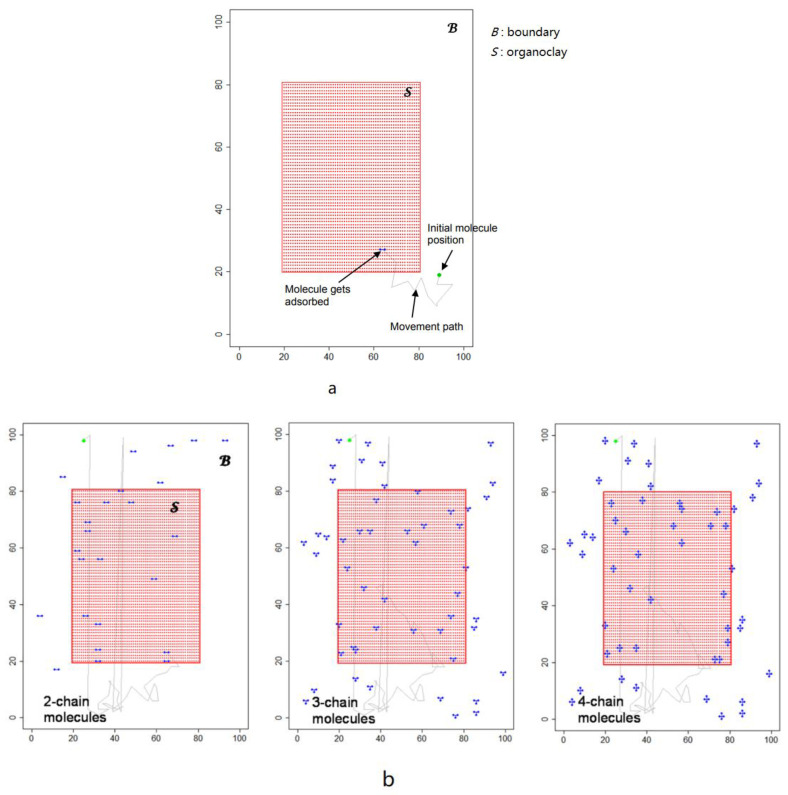
(**a**) The simulation domain with the hypothetical molecule movement path; (**b**) predicted simulation output of different molecule chains [16].

The two factors that have the greatest impact on each dependent variable from the Pareto plot were selected to obtain the response surface (Figure 6). As shown in Figure 6a,b, the response surfaces of plastic viscosity and apparent viscosity are very similar. The viscosity decreases significantly with increasing temperature, whereas the viscosity increases with increasing viscosifier concentration at a fixed temperature. For thixotropy (Figure 6c), it can be seen that this variable increases significantly with increasing temperature. The response surface of yield stress (Figure 6d) shows that the value of this variable increases with temperature [124]. The fundamental phenomenon of nonionic surfactant adsorption on organic bentonite and its impact on the rheology of synthetic drilling fluids were elucidated by molecular descriptor analysis and Monte Carlo simulations. The simulation region is a 100 × 100 grid used as the system boundary, as shown in Figure 7a. Figure 7b shows examples of simulation output for different types of nonionic surfactant molecules. Research shows that understanding the relationship and importance of chemical structure to drilling-fluid properties through machine-learning models can help researchers develop new chemical additives [16].

The significance of molecular theory research on modified montmorillonite for high-performance modified bentonite lies in analyzing the intrinsic mechanism and intermolecular interactions of modified montmorillonite to guide the optimization and modification of bentonite. Firstly, molecular theory can conduct in-depth research on the interaction mechanism between montmorillonite surface modifiers and bentonite through simulation and calculation methods, in order to explore the chemical bonds, adsorption states, and arrangement of modifier molecules on the surface of montmorillonite minerals and further reveal the interaction mechanics between bentonite modifiers, providing theoretical guidance for the design and synthesis of high-performance modified bentonite. Secondly, molecular theory can predict the interaction ability and stability between modifiers and montmorillonite, providing a basis for the selection and design of modifiers. Molecular simulation and calculation can be used to study the physical and chemical properties of the montmorillonite surface, including aspects such as charge distribution, solubility, and adsorption capacity. By simulating the interaction with modifiers, the affinity and stability between modifiers and bentonite can be evaluated, providing important references for selecting suitable modifiers and optimizing modification processes. In addition, molecular theory can provide a deep understanding and explanation of the modification mechanism of montmorillonite. By studying the molecular structure and kinetic characteristics of modified montmorillonite, the basic process and mechanism of montmorillonite modification can be revealed, and the interaction path and energy changes between modifiers and montmorillonite can be further explored, providing theoretical support and guidance for the application of modified montmorillonite.

In summary, molecular theoretical research on modified montmorillonite is of great significance for the study of high-performance modified bentonite. It helps to reveal the internal mechanism of modified bentonite, predict the interaction ability and stability between modifiers and montmorillonite, and provide theoretical guidance for the design, selection, and synthesis of high-performance modified bentonite.

## 6. Conclusions

Bentonite is relatively rich in resources. It can be foreseen that with further research, more modified organic bentonite with novel structures and various types will be synthesized to replace the current traditional drilling-fluid treatment agent products. More “intelligent” drilling fluids will be developed, and the rapid development of efficient and environmentally friendly drilling-fluid technology will be realized.

(1) Intercalation into montmorillonite using the solution or other forces is a widely used synthesis method in the field of composite-materials research. It can significantly improve the performance of the composite material. By reacting the chemical bond with appropriate branched or functional side groups, the formed graft product has become a simple and practical way to expand the application field of polymers and improve the properties of polymer materials. The conditions required for esterification, etherification, and other reactions are relatively complex, but the modification effect of organic bentonite is also obvious, which can be used in fields such as organic synthesis.

(2) Molecular simulation can study processes that are still difficult to study by modern experimental methods, shorten the development cycle, and reduce development costs. In addition, the structure and properties of fluids can be directly observed and studied under micro-restricted conditions through computer simulation. It can be widely used to study the interlayer structure of organic bentonite.

(3) The study of organic bentonite is a large field and shows enormous potential to be explored. Consumption of organically modified bentonite in the drilling industry is expected to increase as the global focus on environmental responsibility continues to increase. Research on nanoscale bentonite composites is expected to accelerate, as materials science increasingly focuses on nanoscale interactions among materials.

(4) Future research should continue to focus on the development of new bentonite materials. Bentonite has several advantages when used as a carrier, including its lack of toxicity, chemical reactivity, and hydrophilicity, which allow easy fixation of biomolecules. While giving full play to the advantages of bentonite, other functional substances can be used to give bentonite new structures and functions. This can not only promote the development of bentonite but also promote the industrial application and promotion of bentonite materials.

## Data Availability

Not applicable.

## Abbreviations

Mt	Montmorillonite
API	American Petroleum Institute
ROP	Rate of penetration
XRD	X-ray diffraction
TGA	Thermogravimetric analysis
CTAB	Hexadecyltrimethylammonium bromide
DTAB	*N*,*N*,*N*-trimethyl-1-dodecanaminium bromide
OTAB	Octadecyl trimethyl ammonium bromide
TTAB	Tetradecyltrimethylammonium bromide
PLSN	Polymer/layered silicate nanocomposites
PAM	Polyacrylamide
CPNm	Clay–polymer nanocomposites
FT-IR	Fourier transform infrared spectrometer
HDTMA	Cetyltrimethylammonium chloride
PPG	Polypropylene glycol
HDTMS	Hexadecyltrimethoxysilane
PAMAM	Amine-terminated polyamidoamine
TEM	Transmission electron microscopy
WBDF	Water-based drilling fluids
PEA	Amino-terminated polyoxypropylene
HAES	Amidosilanols
MPS	Methacryloxypropyltrimethoxysilane
ILs	Green ionic liquids
PLA	Polylactic acid
PS	Polystyrene
SEM	Scanning electron microscopy
PEO	Polyethylene oxide
DSC	Differential scanning calorimetry
PB	Polycationic bentonite
PP	Polypropylene
PP-MA	Maleic anhydride PP
Si-HPEI	Silane coupling agent
HPEI	Hyperbranched polyethyleneimine
Si–SL	Silane coupling agent
SL	Sulfonated lignin
NBR	Nitrile butadiene rubber
KH570	γ-methacryloyloxytrimethoxysilane
PAC	Polyaluminium hloride
SDBS	Sodium dodecylbenzylsulfonate
PVC	Polyvinyl chloride
CMC	Carboxymethyl cellulose
AM	Acrylamide
CMCS	Carboxymethyl chitosan
WBDMS	Water-based drilling mud system
PMAA	Polymethacrylic acid
IR	Infrared spectroscopy
XPS	X-ray photoelectron spectroscopy
AMPS	2-acrylamide-2-methylpropanesulfonic acid
DMDAAC	Diallyldimethylammonium chloride
DMAA	*N*,*N*-dimethyl Acrylamide
SSS	Sodium p-styrene sulfonate
SMP	Sulfobenzaldehyde
MSP	3-vinyltriethoxysilane
CMS	Carboxymethyl starch
CS	Corn starch
CES	Compound etherified starch
